# Laboratory study of *Fritillaria* lifecycle reveals key morphogenetic events leading to genus-specific anatomy

**DOI:** 10.1186/s12983-022-00471-y

**Published:** 2022-10-28

**Authors:** Simon Henriet, Anne Aasjord, Daniel Chourrout

**Affiliations:** Sars International Centre for Marine Molecular Biology, University of Bergen, Bergen, Norway

## Abstract

A fascinating variety of adult body plans can be found in the Tunicates, the closest existing relatives of vertebrates. A distinctive feature of the larvacean class of pelagic tunicates is the presence of a highly specialized surface epithelium that produces a cellulose test, the “larvacean house”. While substantial differences exist between the anatomy of larvacean families, most of the ontogeny is derived from the observations of a single genus, *Oikopleura*. We present the first study of *Fritillaria* development based on the observation of individuals reproduced in the laboratory. Like the other small epipelagic species *Oikopleura dioica*, the larvae of *Fritillaria borealis* grow rapidly in the laboratory, and they acquire the adult form within a day. We could show that major morphological differences exhibited by *Fritillaria* and *Oikopleura* adults originate from a key developmental stage during larval organogenesis. Here, the surface epithelium progressively retracts from the posterior digestive organs of *Fritillaria* larvae, and it establishes house-producing territories around the pharynx. Our results show that the divergence between larvacean genera was associated with a profound rearrangement of the mechanisms controlling the differentiation of the larval ectoderm.

**Supplementary Information:**

The online version contains supplementary material available at 10.1186/s12983-022-00471-y.

## Introduction

Larvaceans, also known as appendicularians, are a class of tunicates characterized by the production of a unique filter-feeding apparatus, nicknamed the “larvacean house”. The house is a complex structure secreted by the animal, which consists in multiple rooms whose function is to size-select food particles (usually microalgae and bacteria) and to channel the water flow in or out from the digestive tract [1]. Discarded houses will sink and aggregate organic matter, thus fast-growing larvacean populations during algal bloom will contribute to the transfer of significant amounts of carbon to the sea floor [2]. While the mechanisms that drive the assembly of the cellulose and protein mesh structure remain largely unknown, some of the anatomical and molecular basis for house synthesis have been uncovered recently—mostly by studying the species *Oikopleura dioica*. Results obtained with this species have revealed important prerequisites for the emergence of house synthesis such as: the acquisition of bacterial cellulose synthase genes [3], the presence of a novel family of proteins—the oikosins [4], and the deployment of novel genetic networks to differentiate cell territories within the house-producing surface organ, also known as the oikoplastic epithelium [5, 6].

Zoological records have permitted to establish three larvacean families, the Oikopleuridae, the Kowalevskiidae, and the Fritillariidae. These organisms have been studied mostly by examining specimens freshly caught in the open ocean and brought aboard exploration vessels, which usually do not offer suitable conditions for reproducing captured specimens and studying lifecycles. Apart from anatomical descriptions—often ancient and produced with rudimentary microscopy techniques, the biology of many larvaceans remains obscure. The few exceptions are found in the Oikopleuridae, and they include *O. dioica*, a coastal species that can be raised in the laboratory [7, 8]. ROVs may grant better access to larvaceans biology in situ, and they have been deployed for studying giant Oikopleuridae off the Northwest American coast [9]. While these recent studies have provided important insights about the evolution and development of larvaceans [10], there is a contrasting lack of knowledge for other families. Modern descriptions of the Kowalevskiidae are scarce [11, 12], and recent studies of Fritillaridae have focused on few biological features only [13–15]. Due to the lack of comparative information, we may under-appreciate the variety of biological processes present in larvaceans, which in turn compromises our understanding of their evolutionary history at the base of the vertebrate lineage [16].

**Fig. 1:**
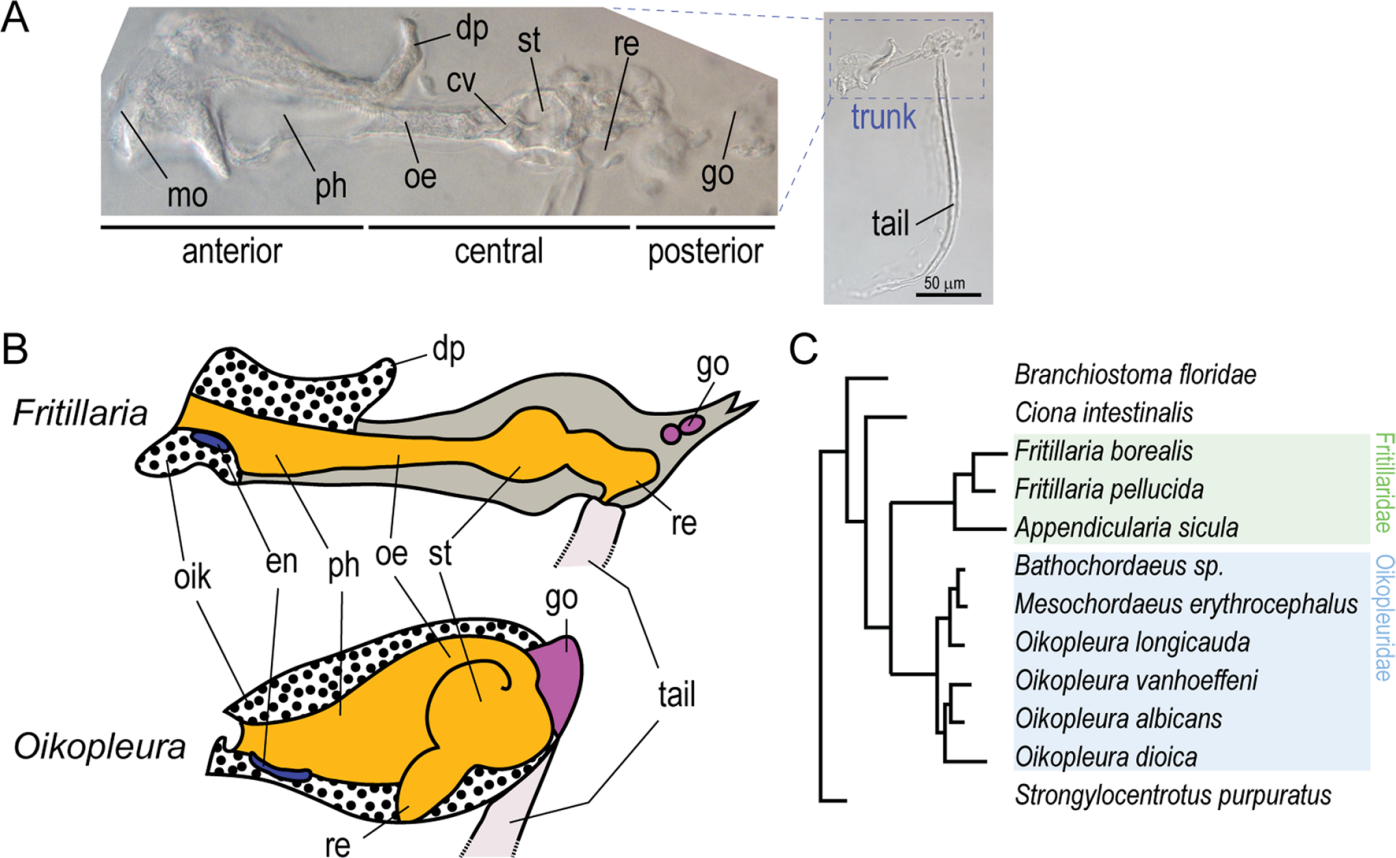
Anatomy of the trunk in two larvaceans families. **A** Juvenile *F. borealis* (2 days post fertilization (dpf)), with detail of the trunk organs. **B** Schematic representation of the trunk organs in *Fritillaria* and *Oikopleura*. **C** Phylogeny of larvaceans adapted from Naville et al*.* [17]. en, endostyle; cv, cardiac valve; dp, dorsal projection over the esophagus; go, gonad; oe, esophagus; oik, oikoplastic epithelium; ph, pharynx; st, stomach; re, rectum

The genus *Fritillaria* contains interesting species to address some of the knowledge gaps. At the ecological level, *Fritillaria borealis* shares similarities with *O. dioica*. These species have overlapping distributions and they are both found in coastal surface waters. At the genetic level, *F. borealis* and *O. dioica* are characterized by small and fast-evolving genomes, a likely result of large effective population size and other mechanisms driving chromosome reduction and rearrangement [5, 17, 18]. However, reproduction modes differ largely since *O. dioica* is the only non-hermaphroditic tunicate. Like all larvaceans, the general anatomy of these two species is relatively simple and consists in a trunk with feeding, reproductive and sensory organs, followed post-anally by a long tail used by the animal for locomotion and to generate water flow in the house (Fig. 1A).

At the end of larval development, the trunk of *Oikopleura* assumes a round compact shape. Its anterior part consists in a cavity that includes all digestive organs, and which is covered with the oikoplastic epithelium [13, 19]. The posterior part contains the reproductive organs, whose size and complexity will increase dramatically during sexual maturation [20]. In *Fritillaria*, the trunk has a very different, elongated shape that can be divided in three segments along the antero-posterior (AP) axis [21, 22] (Fig. 1A). The anterior-most segment consists in a cavity covered with oikoplastic epithelium that contains the mouth, the cerebral ganglion, and the pharynx. It is connected by the esophagus—which consists in a single layer of small cells, to a central segment that contains a simplified gut composed of few large cells [22]. The posterior-most segment corresponds to the gonad sac, which grows inside a cuticular extension of the trunk. Remarkably, there is no epidermis visible in central and posterior segments, and the only barrier to the outer environment is a thin “cuticular layer” without visible cellular structures [22]. *Fritillaria* and *Oikopleura* also exhibit fundamental differences during house production, which could have an impact on their respective contribution to “marine snow” aggregates. In *Oikopleura*, the house is inflated only once after its synthesis, it surrounds the whole animal, and it is frequently discarded (usually following clogging or stress) [23, 24]. In *Fritillaria*, the house expands on the anterior side only, and the animal is capable to deflate and re-inflate the house at regular time intervals, which is believed to facilitate filter function by chasing clogged particles [14].

Taking advantage of a laboratory culture maintained over several weeks, we have gained better insight on some of the known features of *Fritillaria*. These include its distinctive trunk anatomy—previously described in adults only [22], the development of the female gonad—so far documented with low-resolution microscopy techniques [25], and the short lifecycle whose duration was previously inferred from plankton tows [26]. By using confocal microscopy to gain detailed observations of larvae, we could reveal common features also present in *O. dioica*, and we could demonstrate that a key developmental step during organogenesis determines the differences between *Oikopleura* and *Fritillaria* body shape. The mechanisms appear to involve the differentiation of the larval trunk in three distinct regions that are analogous to the segments observed in adult animals. At the same time as the trunk viscera develop, we could observe the progressive dissociation of the larval ectoderm from the posterior end of the trunk, and its differentiation into an oikoplastic epithelium at the anterior end. These morphogenetic processes are not observed in *O. dioica*, and they can clearly account for the organization of digestive organs and house-producing epithelium in the adult form of *F. borealis*.

## Methods

### Acquisition of described species

Seawater samples were collected either from the shore at Espegrend marine station (60° 16′ 10.9″ N 5° 13′ 19.7″ E) by submerging and hoisting 17 L polycarbonate beakers, or at sea in Herdlefjorden (60° 34′ 00.4″ N 5° 02′ 41.8″ E) by conducting horizontal plankton net hauls at 25 to 5 m depth zones. Large individuals captured with plankton nets often have damaged houses and they tend to get stuck at the water surface. To improve survival rates, we employed different mesh size during the summer (150 µm) and the winter (50 µm) season, and we kept tow speed between 0.3 and 0.5 knots. To obtain *Fritillaria borealis*, we organized multiple field collections between October 2019 to February 2020, and between October 2020 to August 2021. Specimens of *Fritillaria haplostoma* were obtained from the shore at Algerøyna (60° 21′ 38.5″ N 4° 57″ 20.6″ E) in December 2021. Specimens of *Appendicularia sicula* were recovered from plankton net hauls, usually together with *F. borealis* during the spring and summer seasons. Specimens of *Oikopleura dioica* were obtained from a laboratory culture established a the Sars Centre [7].

### Laboratory culture of *F. borealis*

After field collection, seawater samples were brought to a 12 °C cooled aquarium in a laboratory facility [7]. During the following ten days, the samples were inspected for *F. borealis*. Even though large variation can be observed in adult body size, we used hallmark characters such as tail morphology, gonad shape, and the presence of cuticular appendages to identify specimens that likely correspond to the form *F. borealis typical* [14, 27]. Genome assembly and phylogeny analyses confirmed that a single species was bred in our laboratory culture [17]. After sorting, similar-sized animals were pooled and transferred into filtered, UV-treated facility seawater containing a diet of either *Micromonas pusilla* (1–3 µm) or *Synechococcus* sp. (1 µm) at a density of 40.10^6^ cells/L.

Laboratory populations were kept at 12 °C in polypropylene beakers containing facility seawater under paddle agitation [7]. At maturation, 30 *F. borealis* were transferred into a spawn beaker containing 3 L seawater and 20.10^6^ cells/L of *M. pusilla* or *Synechococcus*. Animals close to gamete release can be distinguished from immature ones by the bright white coloration of the gonad and by the loss of the house, which is associated with a distinctive “nodding” swimming pattern. Selected adults were visually inspected for the absence of ectoparasites before dilution into the spawn beaker. The following day, juvenile density in the spawn beaker was estimated by counting 10 mL samples under microscope. Animal fecundity was estimated by plotting the normal distribution of the number of offspring produced per adult, based on 44 spawns conducted over 17 weeks. The offspring was diluted to a density between 46 and 66 animals/L and fed with 40.10^6^ cells/L of *M. pusilla* or *Synechococcus*. Animals remain invisible to the naked eye until day two post-fertilization. At this timepoint, animals were fed again with 40.10^6^ cells/L of *M. pusilla* or *Synechococcus* and, in case of low mortality (density reduced by less than half), a 50% dilution was applied to prevent stasis [28]. Five days post-fertilization, animals were diluted to a density between 10 and 14 animals/L and fed with 80.10^6^ cells/L of *M. pusilla* or *Synechococcus*. Gonad maturation usually takes place between six- and seven-days post-fertilization.

### Preparation of specimens for microscopy

To produce *F. borealis* larvae, adults with mature gonads were placed to spawn in a 1 L glass beaker containing facility seawater at 15 °C, under paddle agitation. Larvae were recovered by filtration on a 10 µm cell strainer (pluriSelect), then washed under microscope with facility seawater.

For fluorescence staining, adult and larvae were fixed with 4% PAF (4% paraformaldehyde, 0.1 M, MOPS, 0.5 M NaCl, pH 7.5) 1 h at room temperature, washed extensively in PBSTE (PBS, 0.2% tween-20, 1 mM EDTA) in the presence of 0.1 M glycine and incubated 1 h at room temperature in blocking solution (3% BSA in PBSTE). For cellulose staining, specimens were incubated 3 days at 4 °C in blocking solution in the presence of 2 µg mL^−1^ of a GFP-Carbohydrate Binding Module 3A fusion (GFP-CBM3, NZYtech). CBM3 protein modules have a strong affinity for crystalline cellulose, a component of larvacean houses [29]. Specimens were further incubated 1 h at room temperature in blocking solution supplemented with 1/10000 dilution of AlexaFluor-Phalloidin conjugate (Invitrogen), washed four times 30 min in PBST, and mounted in antifade reagent with DAPI (Slowfade Gold, ThermoFisher). Mounted specimens were observed with the 60 × objective of confocal laser scanning microscopes (Leica TCS SP5 and Olympus FV3000). Depending on the specimen, we imaged between 63 and 138 planes, using step size ranging from 0.3 to 0.5 µm, and a scanning resolution of 1024 × 1024 pixels with an average of two images per plane. We used ImageJ to analyze confocal stacks and Imaris for 3D volume reconstruction. We studied the larval development of *F. borealis* by examining 20 individuals collected before hatching, and 28 individuals collected after hatching. We compared larval development by examining 25 *O. dioica* individuals collected at different time points after hatching. To document gonad development and house-producing epithelium in adults, we examined 21 *F. borealis* individuals, three *F. haplostoma* individuals, and two *A. sicula* individuals.

Live specimens after larval development (one day post fertilization) were observed using the 10 × objective of a Nikon Eclipse E400 microscope. Retraction of the trunk epidermis was observed with the 40 × objective of a Nikon TMS microscope on a live *F. borealis* larva immobilized on a plastic dish and recorded using a Zeiss Axiocam 208. Stage temperature was maintained below 20 °C using a PeCon microscope incubator.

## Results

### Reproduction of *F. borealis* in the laboratory

Seawater samples from field collection were transported and grown in our laboratory facility [7]. During spring and autumn, when population densities are higher [30], it is often possible to identify few *F. borealis* with developing gonads immediately after collection. Water samples collected during these seasons also contain numerous larvae and small juveniles which usually become visible after two days of growth in the lab. These young animals make a larger contribution to the initial increase of individuals in culture, and their presence is a favorable prognostic for maintaining laboratory populations over a longer term.

**Fig. 2:**
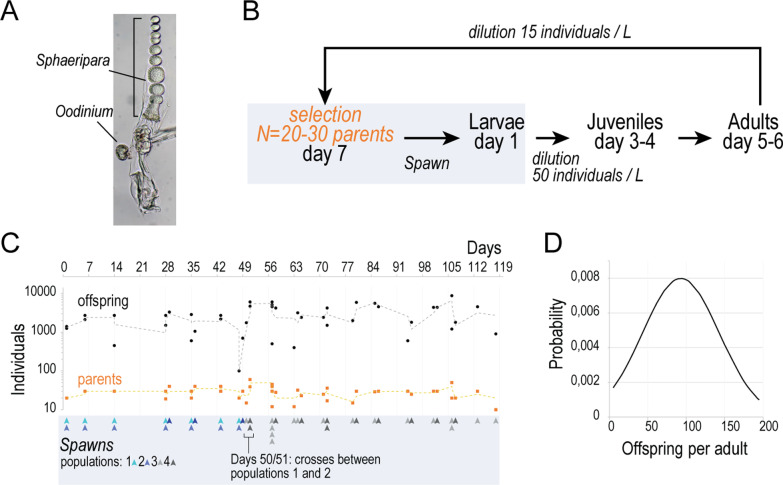
Laboratory culture of *F. borealis*. **A** Wild specimen carrying ectoparasites. **B** Schematic of the procedure for maintaining a laboratory population during the seven days lifecycle. Animal handling steps are indicated with italic font. **C** Animal production in the laboratory during a 17-weeks period. For each spawn conducted (arrowheads), the number of parents (orange squares) and the number of produced offspring (black dots) have been plotted. Curves show 2 periods moving averages for each series. Spawns were established using animals from the same population, excepted at days 50 and 51, when new populations were established by using animals from populations 1 and 2 in the same spawn. **D** Average fecundity of adults determined from the offspring counted after laboratory spawns

Two forms of ectoparasites were found associated with wild specimens (Fig. 2A). The first one is conspicuous on mature animals, as it consists in a row of bead-shaped cells that replace the gonads in the posterior trunk. These parasite cells easily detach from the animal (for example upon transfer to an observation container) and when isolated, they rapidly divide into ciliated swimming forms. The organism is most likely a Syndiniale of the genus *Sphaeripara*. These parasitic dinoflagellates were previously described on *F. pellucida* specimens [31] and their interaction with *F. borealis* was shown indirectly with metagenomics [32]. The second parasite is visible only under microscopy and consists in single, round cells attached to the trunk. It probably corresponds to the dinoflagellate *Oodinium* [33], which we also found on *O. dioica* specimens collected in the same areas.

Several attempts were made to reproduce and maintain the animals over multiple generations. Parasitism was a leading cause of failure, prompting us to systematically screen for non-contaminated animals during the isolation from field sample and reproduction steps. Ciliates can have non-antagonistic relationships with *O. dioica* [34] but in the case of *F. borealis*, we found them more likely to represent a nuisance resulting in clogged houses and delayed growth. We addressed ciliate proliferation by using sterile growth media for the algae cultures used as food source. Feeding *F. borealis* with the pico-algae *Micromonas*, and *Synechococcus* gave the best results, while a diet of larger cells such as the one optimized for *O. dioica* [7] led to poor animal growth, possibly due to inadequate size of food particles [15]. Measures to prevent parasite and ciliate infestation, together with the appropriate diet, have proven decisive to maintain fecundity and to extend the laboratory culture over a longer period. The procedures for handling animals and to reproduce laboratory populations were adapted from Bouquet et al. [7] (Fig. 2B). Weekly spawns were conducted to reproduce the laboratory populations (Fig. 2C) and based on the offspring counted one day after spawn, we estimated the median fecundity at 95 offspring per mature adult (Fig. 2D).

### Germline development and fertilization

**Fig. 3:**
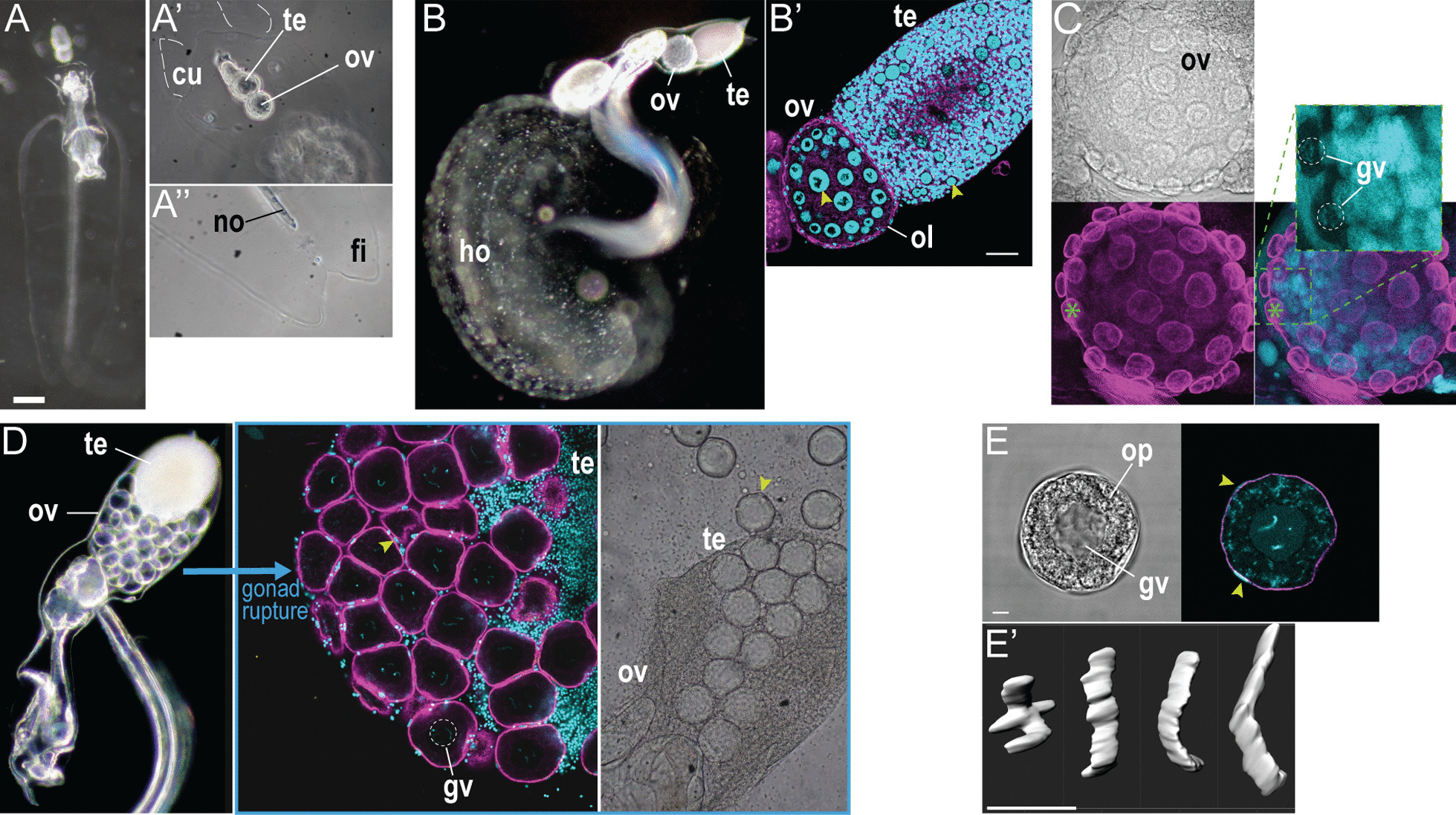
Gonad development in *F. borealis*. **A** Live specimen observed early during the lifecycle (2–3 dpf), with detail of the cuticle around the gonad (**A’**) and at the tip of the tail (**A’’**). Scale bar, 100 microns. **B** Live specimen and fluorescence-stained gonad (**B’**) at an advanced stage of development (5 dpf). Yellow arrowheads indicate nurse nuclei in the ovary and testis. Scale bar, 30 microns. **C** Ovary observed late during the lifecycle (6–7 dpf), showing oocytes growing at the periphery of the ovary (green asterisk). **D** Live specimen before gamete release (7 dpf, left image), and observation of ruptured gonads. At that stage, the ovary cavity consists essentially of mature oocytes, surrounded by reduced nurse nuclei (yellow arrowheads). Gametes are forced through an apical opening of the testis (right image). **E** Oocyte observed immediately after release and 3D reconstruction of the condensed chromosomes present in the germ vesicle (**E’**). Yellow arrowheads indicate reduced nurse nuclei remaining on the oocyte surface. Scale bar, 7 microns. Cyan, DAPI; magenta, phalloidin-AF488; cu, cuticle; fi, tail fin; gv, germ vesicle; ho, house; no, notochord; ol, outer layer of the ovary; op, ooplasm; ov, ovary; te, testis

Larvaceans are semelparous animals that reproduce by external fertilization. In the laboratory, mature founders for the next generation are selected at the end of the seven days lifecycle after examining body size and gonad morphology (Fig. 2D). Two days after fertilization, the gonads of juvenile offspring appear as undifferentiated rudiments surrounded by transparent cuticle (Fig. 3A). The composition of the cuticle is unknown and looks similar to the tail fin. After three to four days of growth, it is possible to distinguish the testis, located in an apical position, and the spherical ovary placed close to the gut. The cuticular extension is completely occupied by gonads at this stage (Fig. 3B), apart from a pair of distal, horn-shaped appendages whose supposed function is to provide anchor points for the house [14]. Based on DAPI staining and previous observations on *O. dioica*, we identified nurse nuclei with high amount of peripheral chromatin [35] (Fig. 3B’). Their size is initially large in immature gonads, and they lie within an extensive actin network reminiscent of the *O. dioica* coenocyst [20]. Like *F. pellucida* [25], female germline nuclei are absent from this network and at later stages, oocytes indeed form an outer layer that surrounds the cavity where nurse nuclei remain (Fig. 3C). Nurse nuclei become dramatically reduced before gamete release, but they remain visible as DAPI-positive material between oocytes (Fig. 3D). Mature oocytes exhibit a prominent germinal vesicle with four chromosomes, a number identical to the *F. pellucida* karyotype [36] (Fig. 3E). Immediately before spawn, the ovary ruptures and oocytes invade the gonad cavity. Gametes are subsequently released in the seawater through an apical opening of the gonad, and during this process the eggs are being forced through the remnants of the testis sac. After oocyte release, DAPI-positive spots can be observed at the surface. Even though we cannot completely exclude that these may correspond to follicular cells, which are frequently encountered in ascidians, the small size and variable number of these spots (between 5 and 12 per egg) also suggest they could represent leftover of nurse cell nuclei stuck to the oocyte.

In ascidians, fertilization is controlled by a non-self/self recognition mechanism that operates at the surface of the egg [37]. Many species are self-sterile, and outcrossing is likely predominant in natural conditions [38]. Self-fertilization of *F. borealis* was never observed in our hands (N = 10), whereas offspring could be recovered from crosses between two individuals at least 60% of the time (N = 10). Development speed during early embryogenesis is comparable to *O. dioica*, with rapid blastomere divisions after fertilization (every 30 min at 15 °C) and hatching of swimming larvae between 4 and 6 h after fertilization.

### Anatomy of the house-producing epithelium

EM sections have offered fundamental insight into the anatomy of the different larvacean families [12, 22, 39], and the simple sample preparation procedure is well-suited for studying species with limited availability. Whereas EM permitted to gain exquisite knowledge about the gut anatomy [12, 22, 40] of *F. borealis*, it brought comparatively little information about the organization of the house-producing epithelium. For observing this organ, techniques that are capable to reveal the arrangement of surface tissues and the filamentous structures present in larvacean houses appear more appropriate.

**Fig. 4:**
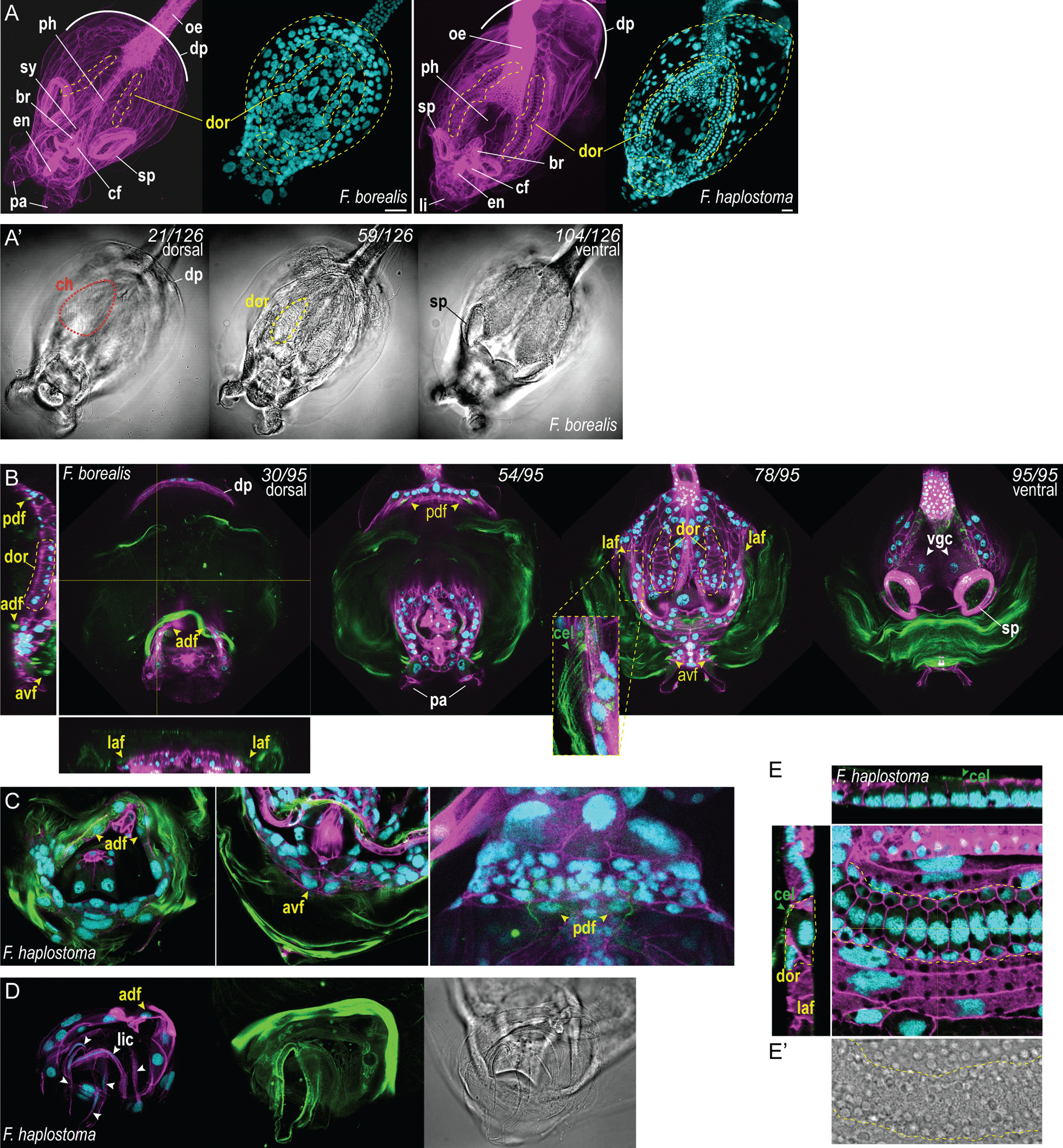
House-producing cells in post-larval stages of *Fritillaria*. **A** Maximum intensity projection of the anterior trunk of fluorescence-stained specimens of *F. borealis* and *F. haplostoma*, showing the viscera and house-producing epithelium (dashed areas). The white arc outlines the dorsal projection of the anterior trunk over the esophagus. Optical sections (**A’**) with bright field illumination show, from the dorsal to the ventral side: a chamber of the house, the dorsal oikoplastic row, and the spiracle opening. **B** Optical sections of the anterior trunk of *F. borealis*. Left and bottom panels show orthogonal projections of the Z-stack. Yellow arrowheads indicate fields of house-producing cells, green arrowheads show cellulose fibrils secreted from the surface of the epithelium. **C** House-producing cells in the anterior trunk of *F. haplostoma*. Like *F. borealis*, cellulose-producing fields are located over and under the mouth (left and middle images), and in the dorsal projection over the esophagus (right image). **D** Cell organization (left image) and cellulose “mask” (middle image) in the lip organ in *F. haplostoma*. **E** High magnification of a dorsal oikoplastic row of cells in fluorescence-stained *F. haplostoma* and bright-field illumination showing the opaque organelles (**E’**). Cyan, DAPI; green, GFP-CBM; magenta, phalloidin-AF488; adf, anterior dorsal oikoplastic field; avf, anterior ventral oikoplastic field; br, brain; ce, cellulose fibrils; cf, ciliary funnel; ch, house chamber; dp, dorsal projection over the esophagus; dor, dorsal oikoplastic row of cells; en, endostyle; laf, lateral oikoplastic field; li, lip; lic, lip cell; oe, esophagus; pa, palps; pdf, posterior dorsal oikoplastic field; ph, pharynx; sp, spiracle; sy, statocyst; vgc, ventral giant cell. Scale bar, 20 microns

We first used a combination of DAPI and phalloidin staining to reveal individual cells in the trunk of *F. borealis*. This approach permitted to easily identify feeding organs and some organs of the nervous systems (Fig. 4A). However, these stains proved unconclusive for attributing house-producing function to specific epithelial cells. Unlike Oikopleuridae, whose epithelium shows well-delimited fields of cells with characteristic shape and size [41], most of the *F. borealis* surface nuclei appear homogenous excepted for a pair of rows of cells flanking the pharyngeal midline, on the dorsal side of the anterior trunk (Fig. 4A). Moreover, the flattened shape of the *Fritillaria* trunk leads to most animals being oriented dorso-ventrally to the objective, which is problematic for distinguishing superficial cells from other tissues located underneath. Confocal scanning proved useful to address this issue, and we used a fluorescent Carbohydrate-Binding Module (CBM) to stain cellulose and better identify cells that contribute to house production.

Cellulose fibrils originate from different locations on the surface of the anterior trunk. Many cellulose-secreting cells are in the dorsal projection of the epidermis that extends laterally and dorsally over the oesophagus (Figs. 1A, 4A, B). Compared to other cells at the surface, those are significantly larger, and they remind us of the giant cells of the *Oikopleura* epithelium [42]. Fritillaridae and Oikopleuridae may share conserved mechanisms to increase cell size and ploidy in specific territories of their oikoplastic epithelium [43]. Cellulose fibrils also originated from the dorsal and ventral epidermis around the mouth, giving clear indication for the presence of oikoplastic epithelium. Even though we could not distinguish cellulose staining on the pair of dorsal rows of cells, their location strongly suggest they contribute to the formation of specific structures in the house, like a pair of dorsal chambers seen in houses that have not yet been inflated (Fig. 4A’). This group of cells will be subsequently referred to as “dorsal oikoplastic rows”. Outside the spiracle opening and the anterior house-producing fields, the ventral side appears extremely simplified and most of its surface is occupied by two giant cells whose function is unknown (Fig. 4B).

We conducted the same observations on mature individuals from another species of Fritillaridae collected in the same area. Based on spiracle, tail and testis morphology (Additional file 1: Fig. S1) [21, 27, 44] we identified the specimens as *Fritillaria haplostoma*. However, while its position relative to the male gonad is consistent with former descriptions of *F. haplostoma*, we could observe that the female gonad consists in multiple small spherical ovaries (Additional file 1: Fig. S1A), whose structure is identical to the single ovary of *F. borealis* (Fig. 3C). As this was not reported previously, we cannot exclude that our specimens correspond to a new species closely related to *F. haplostoma*. House-producing cell fields were found at conserved locations on the pharyngeal trunk (Fig. 4C), and the specimens showed cellulose fibrils originating from the dorsal oikoplastic rows (Fig. 4E), thus confirming their role in house secretion. We also observed fundamental differences with the anatomy of *F. borealis*, possibly related to specific house structure and feeding behavior. For example, the pair of lateral palps flanking the *F. borealis* mouth is absent in *F. haplostoma*. Instead, we observed a delicate arrangement of cellulose-rich, flattened cells forming a protruding structure around the mouth opening which could corresponds to the “lip” mentioned in previous descriptions [21] (Fig. 4A, D). These mouth appendages are likely to play different functions during feeding, possibly for processing specialized diet. In most cases, we could not compare the amounts of house-producing cells and their morphology with *F. borealis*, the reason being the uncertainty about the maturity stage of *F. haplostoma* specimens, which have been observed immediately after field collection. However, we could observe that the dorsal oikoplastic rows in *F. haplostoma* assumed a more complex arrangement, which consists in two rows of small cells flanking a central row of larger, rectangular cells (Fig. 4A, E). We also observed numerous, large opaque vesicles that are distributed uniformly inside cells across the pharyngal trunk epithelium (Fig. 4E’). These organelles, which were not observed in *F. borealis*, could participate to epithelium function, like secretion of house components or protection against UV radiation.

### Morphogenesis of the trunk epithelium

All larvaceans produce filter-feeding houses, but mechanisms that control the development of house-producing organs remain largely uncharacterized outside *O. dioica*. In this species, epithelium morphogenesis takes place early during development, several hours before the first house is synthesized [6]. While such timing could be conserved during the development of *F. borealis*, the distinct oikoplastic fields we observed in adult animals are strong indications for the presence of different developmental mechanisms. We decided to gain better insight by examining the epithelium of *F. borealis* larvae. We could not acquire live recording of developing embryos, as in vitro fertilized oocytes frequently aborted development when left in vessels without water flow. This proved problematic to study development during blastula and gastrula stages and instead, we focused our study on hatched embryos and swimming larvae recovered from spawn vessels under paddle agitation. The developmental progression of these animals could be determined a posteriori under microscope, by examining key features such as cell number, notochord growth and epithelium patterning.

**Fig. 5:**
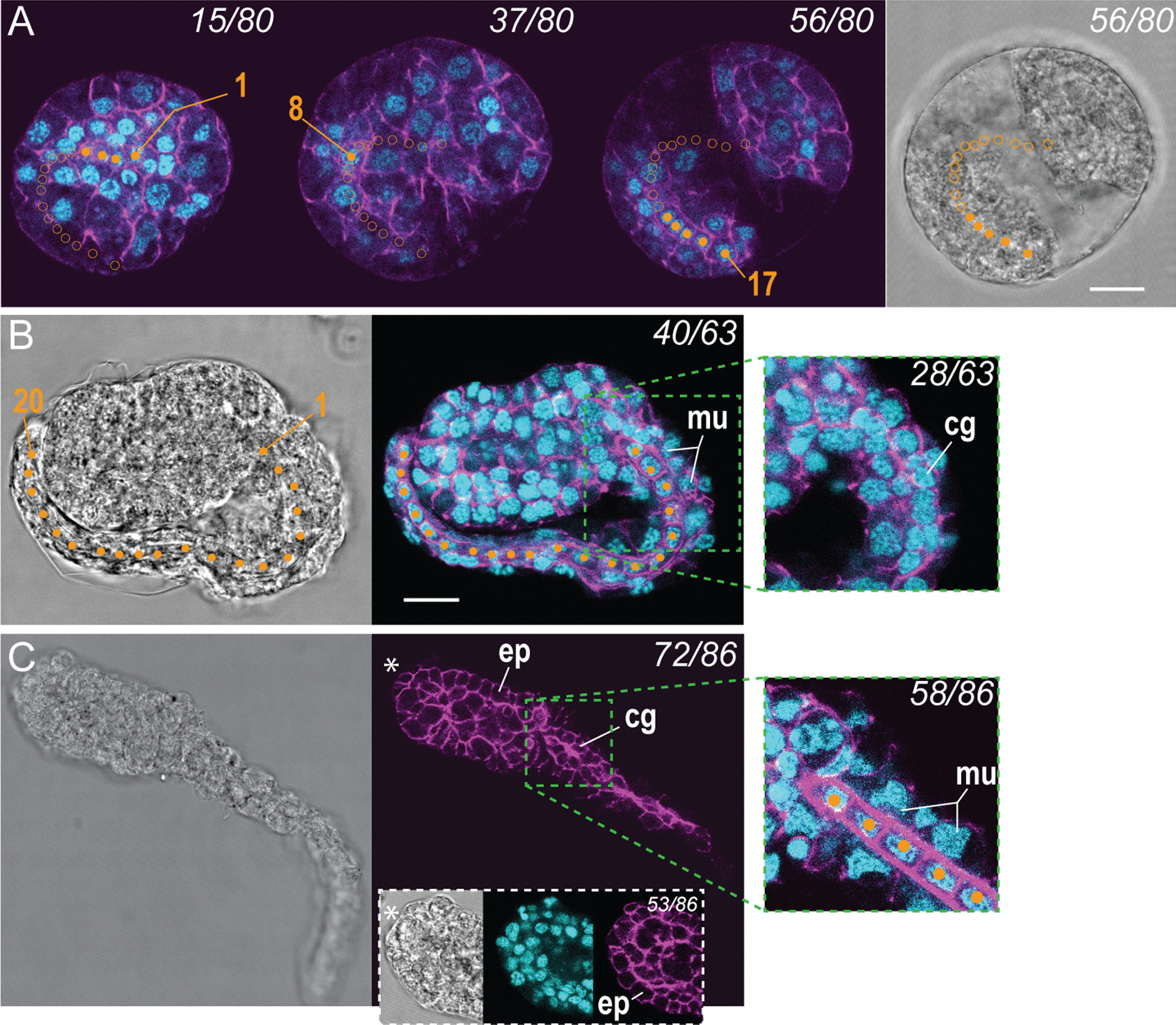
Early stages of notochord and tail development in *F. borealis*. **A** Optical sections showing an embryo at the tailbud stage, with notochord cells stacked along the AP axis (orange dots). Full dots mark notochord nuclei, open dots mark nuclei visible in other sections. **B** Embryo at the end of notochord cell divisions. The closeup shows the caudal ganglion primordium. **C** Embryo immediately after hatching. Closeups show the anterior end of the trunk with the mouth primordium (white asterisk) and detail of the tail with notochord and muscle cells. cg, caudal ganglion; ep, epidermis; mu, muscle cells. Scale bar, 10 microns

Trunk and tail primordia can be recognized before hatching, when the embryo assumes a coiled shape with two lobes that resembles the tailbud stage of *O. dioica* [10] (Fig. 5A). In these animals, the notochord is already visible and consists in 12 to 17 cuboid cells stacked along the AP axis. In the next stage, observed animals are still surrounded by chorion, but the tail and the trunk are more differentiated (Fig. 5B). The notochord cells have completed their divisions, reaching a final number of 20 cells (including a round-shaped terminal cell in the tip of the tail), equal to the *O. dioica* notochord [10]. At that stage, we can easily discern broad muscle cells flanking the notochord, and a small group of cells located on one side of the tail surface that will later give rise to the caudal ganglion. Other cells will give rise to structures such as tail epithelium and the finlets, which are better observed in older larvae (Additional file 1: Fig. S2). Tissues of the trunk remain hard to characterize before hatching, apart from surface columnar cells that will later give rise to the epithelium.

**Fig. 6:**
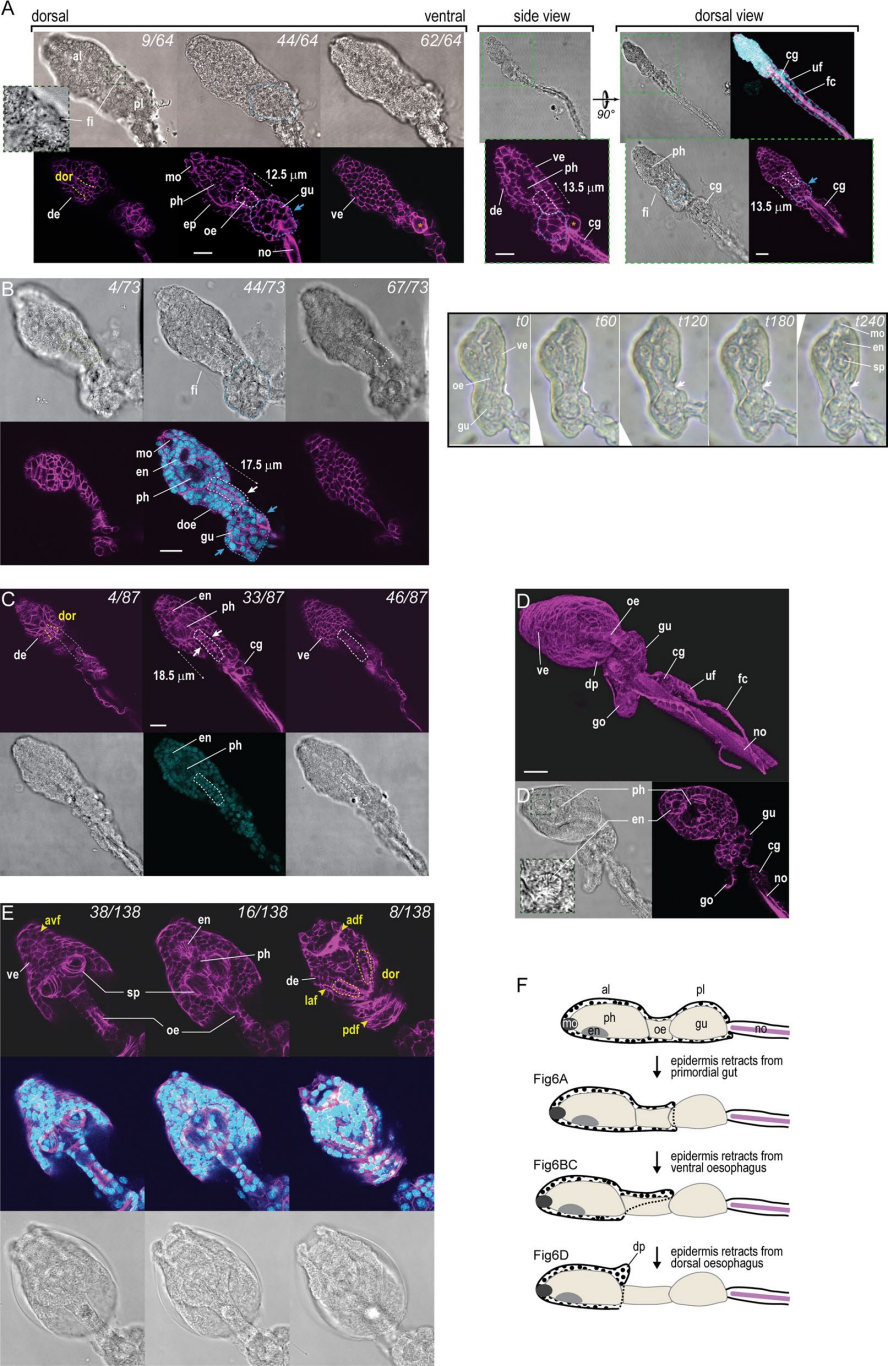
Morphogenesis of the trunk in *F. borealis*. **A** Optical sections on the dorsoventral axis, side view and dorsal view of hatched larvae observed during the first step of epidermis retraction. Note the thickened basal lamina stained with phalloidin (magenta), which outlines the epidermal layer. White and blue dashed lines mark the position of developing esophagus and gut, respectively. Blue arrows show area of the gut where epidermis has retracted. The orange asterisk marks a large cell located on the ventral side which is used to orient the larva. **B** Optical sections (left panel) and movie frame shots (right panel) of larvae observed during the second step of epidermis retraction. At this stage, the epidermal layer (continuous white line in movie frame shots) progressively retracts towards the anterior, leaving the ventral side of the esophagus exposed (white arrows). **C** Optical sections of a larva observed at the end of the second step of epidermis retraction. At this stage, the retraction of the epidermal layer is visible on the left and right sides of the esophagus and progresses towards the dorsal side (white arrows). **D** 3D reconstruction and optical section (**D’**) of a larva during the third step of epidermal retraction. The epidermis has formed a projection over the dorsal side of the esophagus. The gonad primordium is now visible in the posterior trunk, lined by a cuticular material similar to the tail fin. **E** Juvenile with a pre-house. In this animal collected within 24hpf, it is possible to recognize the major anterior organs and house-producing fields of cells on the trunk epidermis. adf, anterior dorsal field; avf, anterior ventral field; laf, lateral field. **F** Schematic description of the epidermis retraction during larval development. al, anterior lobe of the trunk; cg, caudal ganglion; de, dorsal epidermis; doe, dorsal side of the esophagus; dor, dorsal oikoplastic row of cells; dp, dorsal projection; ep, epidermis; en, endostyle; fi, surface fibers; fc, fin cell; go, gonad; gu, gut; mo, mouth; no, notochord; oe, esophagus; ph, pharynx; pl, posterior lobe of the trunk; sp, spiracle; uf, upper fin cell; ve, ventral epidermis. Scale bar, 10 microns

The larvae of *F. borealis* and *O. dioica* look similar immediately after hatching (Fig. 5C). Later, at a stage where organs appear visible in the trunk under bright field microscopy, strong differences appear (Fig. 6A). In *F. borealis*, the trunk differentiates in two lobes that will respectively form the pharynx and the gut. These lobes are separated by a constriction that corresponds to the developing esophagus. The segmentation of the larval trunk, and its elongated growth along the AP axis thus represent the first morphogenetic processes accounting for the characteristic adult morphology (Fig. 1). In contrast, *O. dioica* larvae observed at a similar stage have a compact trunk, where the presumptive gut folds back towards the anterior and develops ventrally under the esophagus (Additional file 1: Fig. S3). We observed intriguing surface fibrils running between the anterior and posterior lobes on the dorsal side of *F. borealis* larvae (closeup of the dorsal side, Fig. 6A), which indicate that epithelial cells are already differentiated and carrying out secretion functions. Accordingly, distinctive cell arrangements also seen in house-producing animals (Fig. 4B) can be recognized on the dorsal epithelium of the anterior lobe of the larva (Fig. 6A) and on the ventral epithelium located under the mouth opening. The function of surface fibrils secreted by the ectoderm at this larval stage remains unknown. We speculate it could participate to the synthesis of the first house rudiment, and it could also contribute to the formation of the “cuticular layer” [45] present around the posterior trunk in post-larval stages (Fig. 2).

In *F. borealis* and in *O. dioica* as well, the larval epithelium initially covers all inner tissues of the trunk, while surface cells located at the anterior end start to develop mouth structures (closeup in Figs. 5C, 6A, Additional file 1: Fig. S3). The inner tissues of the trunk will remain covered throughout the larval development of *O. dioica*, excepted at its posterior end where germline precursors will later give rise to the gonad (Fig. 1), and at the spiracle and anal openings. By comparing hatched larvae collected at successive developmental stages, we could show that in *F. borealis* the trunk epithelium progressively retracts from the posterior end towards the anterior, leaving exposed the inner tissues (Fig. 6). We distinguished three steps during this process. During the first step, retraction of the epithelium from the posterior trunk (Fig. 6A) appears to coincide with the elongation of the esophagus along the AP axis (which could be driven by the proliferation of esophageal cells), and it concludes with the complete absence of outer cell layer around the gut primordia (blue arrows, Fig. 6B). The second step was observed when the endostyle and the pharynx have acquired their characteristic morphology in the anterior trunk. Here, the ectoderm dissociates progressively from the esophagus and leaves the ventral side first (white arrow, Fig. 6B). A multi-layered mass of cells is still visible on the dorsal side (annotation “doe”, Fig. 6B), suggesting that the retraction could be driven by the collective migration of ectodermal cells. At the end of the second step, the esophagus has lost all cell-to-cell contacts with the epithelium, but it remains flanked by epithelial tissue (white arrows, Fig. 6C). During the third step the epithelium forms the dorsal projection over the esophagus (Fig. 6D), where house producing cells were shown earlier (Fig. 4A, B). On the ventral side, the number of epithelial cells is dramatically reduced after the spiracles have appeared, suggesting that some of them could contribute to spiracle formation (Fig. 6E).

## Discussion and conclusion

Tunicates are a fascinating group of animals with considerable diversity in size, feeding strategy and reproduction. However, biological studies have been more sustained on sessile tunicates (represented by solitary and colonial ascidians) than on the pelagic ones, even though the latter are increasingly recognized as key components of the oceanic ecosystems [46]. Many pelagic tunicates are difficult to access and as a matter of fact, the abundant and ubiquitous *O. dioica* rapidly became one the few species to be established in a continuous laboratory culture [7]. Studying the *O. dioica* genome alone revealed surprising features and illuminated some of the molecular bases underlying the biology of this species [5]. However, comparative approaches based on a variety of genomes [17] have proven more adequate to uncover general mechanisms responsible for the evolution of larvaceans. Under this perspective, bringing *F. borealis* to the laboratory represents a decisive step for testing the conservation of biological processes between larvaceans families. By significantly improving the level of detail of the *Fritillaria* anatomy, our study provides original results that will be useful for future comparative studies aiming at understanding the evolution, the development, and the function of the larvacean house.

The production and the structure of filter-feeding houses can vary widely between larvacean families [14], and also between species that belong to the same genus [47]. How these differences could impact animal growth and reproductive success remain largely speculative. For instance, it can be considered that feeding in oikopleurids is less cost-effective, since a fraction of the resources extracted during grazing is immediately spent for the renewal of discarded houses. This hypothesis would be best addressed by counting the number of houses produced per individuals in *F. borealis* and *O. dioica*. Generation times measured in our laboratory culture are equivalent for *O. dioica* and *F. borealis*, suggesting that feeding behavior does not have a strong influence on development speed, at least when animals are raised at comparable densities. Outside stasis conditions [28], the duration of lifecycle could be controlled by parameters that have proximal effects on growth and maturation (for example metabolic rate), or by some parameters with more indirect effects such as body size and genome content. Gaining access to the complete lifecycle of other species with genomes [17] larger to *O. dioica* and *F. borealis* could bring interesting insights to this question.

Descriptions of the house-producing epithelium have been reported early in the genus *Oikopleura* [48, 49] and *Fritillaria* [21, 50]. However, prior to the present study, modern microscopy techniques were employed mostly with *Oikopleura*, where comparison of species showed that conserved fields of oikoplastic cells [41] formed a characteristic pattern at the surface of the trunk. Our results also show that distinctive, conserved cell arrangements are present in the *Fritillaria* epithelium, with the most conspicuous one being the dorsal oikoplastic rows of cells. This cell arrangement was readily visible in our specimens and it could also be recognized in ancient zoological drawings of *F. venusta* and *F. ritteri* [44, 51]. Genus-specific arrangements of house-producing cells immediately raise questions about the evolution of the epithelium in larvaceans and the nature of the oikoplastic organ in the common ancestor. While the species *Appendicularia sicula* lacks the characteristic trunk anatomy of the *Fritillaria* genus, we have observed similar features at the surface epithelium of the pharyngeal trunk, such as dorsal rows of cells close to the midline (Additional file 1: Fig. S4). Although the number of cells present in these structures is considerably reduced compared to *Fritillaria*, their location suggest that arrangements of house-producing cells could be conserved between different genus within the Fritillaridae. We found at least one *CesA* gene [3] in the *F. borealis* genome, but we could not clearly detect any orthologue of the oikosins that have been characterized in *O. dioica* [52]. It suggests that the gene complement necessary for epithelium function may have diverged significantly between larvacean families. In the future, examining the expression of transcription factors involved in the patterning of the trunk epithelium [6] could bring better insight for understanding how this organ evolved in different families of larvaceans.

Although larvaceans embryos can be recovered from seawater samples, producing them from adults that have been isolated and maintained in culture presents several advantages. It allows the examination of a larger number of specimens at different stages of the lifecycle, and it also helps to dissipate uncertainties about the species to which the embryos belong. The latter point is of critical importance since larvae of *Fritillaria* and *Oikopleura* can appear very similar when examined superficially. To our knowledge, we first provide detailed observations of the *Fritillaria* larva, revealing developmental processes that are key for understanding differences between larvacean families. However, gaining access to morphogenetic events and gene expression during the development represent future milestones that will be essential for exploring some of the questions raised by our study. The results suggest indeed that specific decisions controlling trunk anatomy are established early during development, and they lead to differences already visible after hatching; with the *F. borealis* larvae having a slightly more elongated shape than *O. dioica* (Fig. 5C).

The first descriptions of the *Fritillaria* genus already reported that, unlike *Oikopleura*, the house-producing epithelium is restricted to the anterior part of the trunk [21]. It was shown more recently that the epidermis is completely absent in the posterior trunk, and digestive organs are in fact separated from the environment by a thin acellular layer (also described as a “cuticular layer” by some authors) [22, 53]. This outer layer was difficult to observe in our specimens and no indications exist so far about its constitutive material. We suppose that such structure should act as a simplified epidermis that provides fundamental functions like barrier to the external environment and osmoregulation. In principle, it should be anchored to the outer surface of the digestive tract by the extracellular matrix, whose components could also participate to protective and biochemical functions. While the exact origins of the surface layer remain to be determined, the results of our study suggest that it is established during the retraction of the ectoderm from the posterior trunk of the larva. Future investigations of the development and anatomy in Fritillaridae will certainly benefit to understanding how such striking differences have emerged during evolution. For that purpose, live imaging of developing embryos and ultrastructural approaches based on EM could bring key information about the composition of the animal’s surface and its physiological roles.

## Supplementary Information

**Additional file 1: Fig. S1.** Identification of *F. haplostoma*. A) Observation of specimen with hallmark characters for species determination. B) Simplified species determination key for the *Fritillaria* genus, adapted from Fenaux [27]. ac, amphichordate cells; ol, outer layer of the ovary; ov, ovary; sc, subchordate cells; te, testis. **Fig. S2.** Tail development in *Fritillaria borealis*. A) Optical sections in the tail of the early larva (same specimen shown in Fig. 6A), showing the arrangement of different cell types around the notochord. Bottom panels show orthogonal projections at three positions along the tail. B) Optical sections in the tail of the early larva (same specimen shown in Fig. 6D). cg, caudal ganglion; dp, dorsal projection over the oesophagus; fc, fin cell; fl, finlet; go, gonad; gu, gut; mo, mouth; mu, muscle cell; ne, nerve cell; no, notochord; oe, esophagus; te, tail epithelium cell; uf, upper fin cell; ve, ventral epithelium of the trunk. Scale bar, 10 microns. **Fig. S3.** The larval trunk of *O. dioica*. Optical sections of fluorescence-stained larva at 8 h post-fertilization, showing the epidermis covering the entire viscera and the location of trunk organs. Yellow, white and blue dashed line respectively outline the house-producing epithelium, the esophagus, and the gut. ar, anterior rosette; en, endostyle; ep, epidermis; fo, field of Fol; gu, gut; oe, esophagus; mo, mouth; ph, pharynx; ve, ventral epithelium. **Fig. S4.** House-producing cells in *Appendicularia sicula*. The specimen, collected from the wild, displays the characteristic blind gut and rectum hypertrophy [40]. Fluorescent staining reveals house-producing fields (dashed areas and arrows) at the surface of the pharyngeal trunk. adf, anterior dorsal oikoplastic field; dor, dorsal oikoplastic row of cells; go, gonads, laf, lateral oikoplastic field; mo, mouth; ph, pharynx; re, rectum.

## Data Availability

The datasets used and/or analysed during the current study are available from the corresponding author on reasonable request.
