# Supplementary material for: Laboratory study of Fritillaria lifecycle reveals key morphogenetic events leading to genus-specific anatomy
Source: Front Zool. 2022 Oct 28;19:26. doi: 10.1186/s12983-022-00471-y (PMC9617304; doi:10.1186/s12983-022-00471-y)
Supplement: Supplementary file 1 — Additional file 1: Fig. S1. Identification of F. haplostoma. A) Observation of specimen with hallmark characters for species determination. B) Simplified species determination key for the Fritillaria genus, adapted from Fenaux [27]. ac, amphichordate cells; ol, outer layer of the ovary; ov, ovary; sc, subchordate cells; te, testis. Fig. S2. Tail development in Fritillaria borealis. A) Optical sections in the tail of the early larva (same specimen shown in Fig. 6A), showing the arrangement of different cell types around the notochord. Bottom panels show orthogonal projections at three positions along the tail. B) Optical sections in the tail of the early larva (same specimen shown in Fig. 6D). cg, caudal ganglion; dp, dorsal projection over the oesophagus; fc, fin cell; fl, finlet; go, gonad; gu, gut; mo, mouth; mu, muscle cell; ne, nerve cell; no, notochord; oe, esophagus; te, tail epithelium cell; uf, upper fin cell; ve, ventral epithelium of the trunk. Scale bar, 10 microns. Fig. S3. The larval trunk of O. dioica. Optical sections of fluorescence-stained larva at 8 h post-fertilization, showing the epidermis covering the entire viscera and the location of trunk organs. Yellow, white and blue dashed line respectively outline the house-producing epithelium, the esophagus, and the gut. ar, anterior rosette; en, endostyle; ep, epidermis; fo, field of Fol; gu, gut; oe, esophagus; mo, mouth; ph, pharynx; ve, ventral epithelium. Fig. S4. House-producing cells in Appendicularia sicula. The specimen, collected from the wild, displays the characteristic blind gut and rectum hypertrophy [40]. Fluorescent staining reveals house-producing fields (dashed areas and arrows) at the surface of the pharyngeal trunk. adf, anterior dorsal oikoplastic field; dor, dorsal oikoplastic row of cells; go, gonads, laf, lateral oikoplastic field; mo, mouth; ph, pharynx; re, rectum. [file 12983_2022_471_MOESM1_ESM.pdf]

A

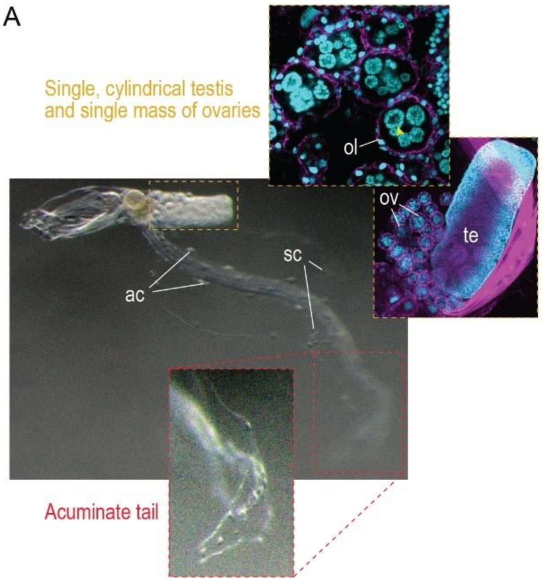

B

Species	Spiracles shape	Distal extremity of the tail	Amphichordate cells	Testis shape	Number of testes
Fritillaria abjornseni	Circular	Acuminate	no	Cylindrical	Single
Fritillaria formica	Circular	Acuminate	no	Triangular	Single
Fritillaria haplostoma	Circular	Acuminate	yes	Cylindrical	Single
Fritillaria arafoera	Circular	Acuminate	no	Triangular	Single
Fritillaria gracilis	Circular	Acuminate	no	C-shaped	Single
Fritillaria polaris	Circular	Acuminate	yes	Ring-shaped	Single
Fritillaria borealis	Circular	Notched	no	Triangular	Single
Fritillaria megachile	Circular	Notched	yes	Cylindrical	Single
Fritillaria pellucida	Circular	Notched	yes	R-shaped	Single
Fritillaria venusta	Circular	Notched	yes	Trapezoidal	Single
Fritillaria pacifica	Oval	Acuminate	yes	Tubular	Single
Fritillaria fraudax	Oval	Acuminate	no	Ribbon	Single
Fritillaria aequatorialis	Oval	Acuminate	no	Ovoid	Single
Fritillaria helenae	Oval	Acuminate	no	Horsehoe	Single
Fritillaria drygalskii	Oval	Acuminate	no	Trapezoidal	Single
Fritillaria aberrans	Slot	Acuminate	no	Cylindrical	Single
Fritillaria fagei	Slot	Acuminate	no	Ovoid	Single
Fritillaria charybdae	Slot	Acuminate	no	Plate	Single
Fritillaria urticans	Slot	Acuminate	no	Plate	Single

Figure S1: Identification of *F. haplostoma*. **A)** Observation of specimen with hallmark characters for species determination. **B)** Simplified species determination key for the *Fritillaria* genus, adapted from Fenaux[1]. ac, amphichordate cells; ol, outer layer of the ovary; ov, ovary; sc, subchordate cells; te, testis.

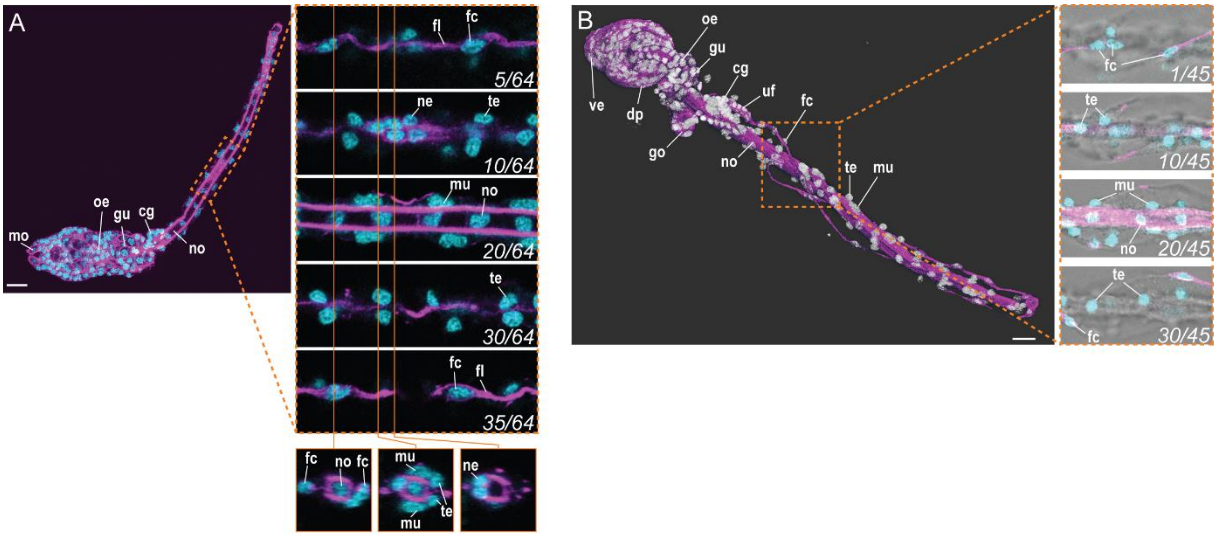

Figure S2: Tail development in *Fritillaria borealis*. **A)** Optical sections in the tail of the early larva (same specimen shown in **Figure 6A**), showing the arrangement of different cell types around the notochord. Bottom panels show orthogonal projections at three positions along the tail. **B)** Optical sections in the tail of the early larva (same specimen shown in **Figure 6D**). cg, caudal ganglion; dp, dorsal projection over the oesophagus; fc, fin cell; fl, finlet; go, gonad; gu, gut; mo, mouth; mu, muscle cell; ne, nerve cell; no, notochord; oe, esophagus; te, tail epithelium cell; uf, upper fin cell; ve, ventral epithelium of the trunk. Scale bar, 10 microns.

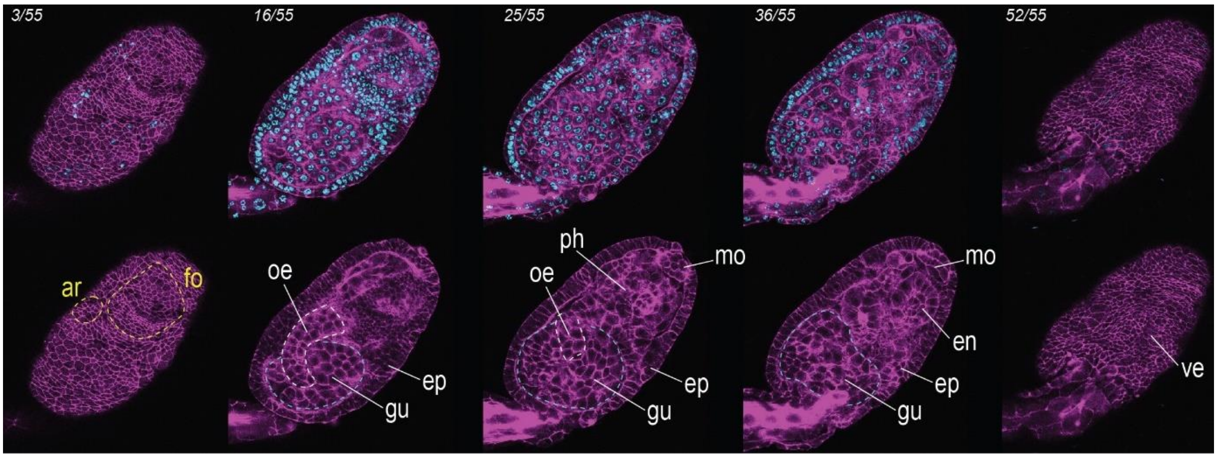

Figure S3: The larval trunk of *O. dioica*. Optical sections of fluorescence-stained larva at 8h post-fertilization, showing the epidermis covering the entire viscera and the location of trunk organs. Yellow, white and blue dashed line respectively outline the house-producing epithelium, the esophagus, and the gut. ar, anterior rosette; en, endostyle; ep, epidermis; fo, field of Fol; gu, gut; oe, esophagus; mo, mouth; ph, pharynx; ve, ventral epithelium.

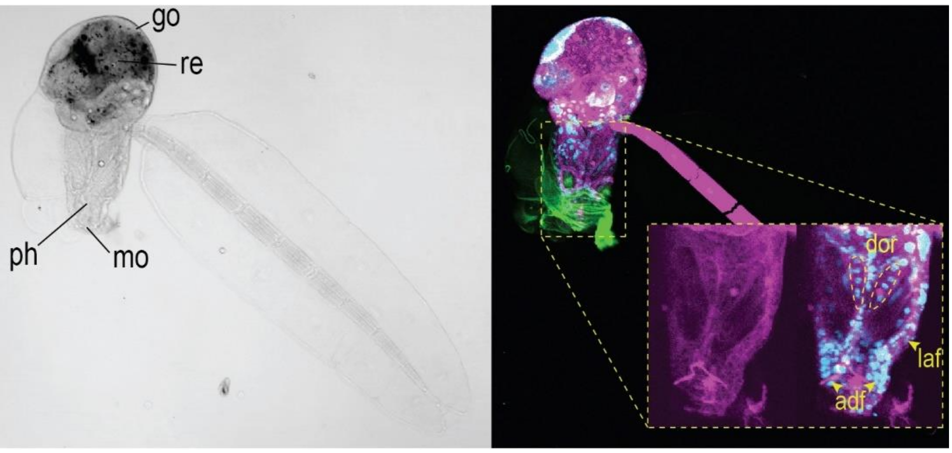

Figure S4: House-producing cells in *Appendicularia sicula*. The specimen, collected from the wild, displays the characteristic blind gut and rectum hypertrophy[2]. Fluorescent staining reveals house-producing fields (dashed areas and arrows) at the surface of the pharyngeal trunk. adf, anterior dorsal oikoplastic field; dor, dorsal oikoplastic row of cells; go, gonads, laf, lateral oikoplastic field; mo, mouth; ph, pharynx; re, rectum.

1. Fenaux R: **Life history of the Appendicularia.** In *The Biology of Pelagic Tunicates.* Edited by Bone Q: Oxford University Press; 1998: 151-159
2. Brena C, Cima F, Burighel P: **The exceptional "blind" gut of Appendicularia sicula (Appendicularia, Tunicata).** *Zoologischer Anzeiger* 2003, **242:**169-177.
